# Comparative Study of the Effects of 1% Atropine on the Anterior Segment

**DOI:** 10.1155/2020/5125243

**Published:** 2020-09-28

**Authors:** Yue Zhou, Xiao Bo Huang, Qi Cai, Jun Jie Li, Yao Jia Xiong, Ye Xun Gong, Li Wei Lin, Yan Zhu, Zhi Min Sun

**Affiliations:** The Second Affiliated College of Nantong University, Nantong, China

## Abstract

**Purpose:**

To investigate the influences of atropine on changes in anterior segment geometry, as measured by ultrasound biomicroscopy in children.

**Methods:**

A prospective observational study was performed. Anterior segment parameters were obtained by UBM before and after the instillation of 1% atropine. Univariate linear regression was performed to identify the variables contributing to the changes in the trabecular meshwork-iris angle (TIA).

**Results:**

The study included 21 boys and 37 girls with a mean age of 10.79 ± 2.53 years. Anterior chamber parameters including the central anterior chamber depth, TIA, angle opening distance at 500 *μ*m from the scleral spur, iris thickness 750 *μ*m and 1500 *μ*m from the scleral spur, trabecular-ciliary angle (TCA), trabecular-ciliary process distance, sclera-iris angle (SIA), and sclera-ciliary process angle significantly increased after cycloplegia (*P* < 0.05). In contrast, the lens vault, iris cross-sectional area, and maximum ciliary muscle thickness significantly decreased after cycloplegia. Univariate analysis identified the change in TCA and the change in SIA and the TIA before mydriasis as determinants of the change in TIA.

**Conclusions:**

Atropine causes statistically significant changes in various anterior segment parameters in children. The change in anterior chamber angle is associated with the change in TCA and the change in SIA and the TIA before mydriasis.

## 1. Introduction

Myopia is a substantial public health problem that has especially affected children in Asia in recent decades [1–3]. In 2050, 49.8% of all people are expected to be myopic [4]. Thus, preventing the development of myopia would strongly affect public health.

Recent studies have suggested that atropine shows promise in myopia control [5–7]. A recent meta-analysis examining interventions for myopia control has reported that high-dose atropine (1% and 0.5%) is significantly superior to other interventions such as orthokeratology, peripheral defocus modifying contact lenses, and specially designed spectacle lenses [8].

In fact, topical atropine has been widely used in clinical settings for myopia control and visual examination in schoolchildren [5, 6, 8–10]. However, the effects of atropine on anterior chamber parameters have not been sufficiently investigated, especially in pediatric eyes. Although angle closure is rare in children and young adults [11], some controversies also exist regarding the changes in anterior chamber parameters after cycloplegia, especially regarding measurement of the anterior chamber angle [12–14]. Therefore, our study aimed to investigate how the angle behaves in response to atropine without the confounding factor of cataracts in children in the period of myopia development. Detailed evaluation of anterior segment parameters may provide important information for understanding ocular pharmacokinetics and aqueous humor dynamics, and for performing intraocular lens calculation, diagnosis, and follow-up in ophthalmic patients. Therefore, studies on the effects of atropine on the biometry parameters are urgently needed, and the results must be interpreted with caution.

The objective of this prospective study was twofold: (1) to quantify cycloplegia-induced changes in ocular parameters in children and (2) to assess changes in the anterior chamber angle and identify relevant influencing factors.

## 2. Materials and Methods

A prospective observational study was performed at the Second Affiliated College of Nantong University, Jiangsu Province, China, between April 2019 and September 2019. Changes in the anterior segment of the eye were examined with ultrasound biomicroscopy (UBM). All study procedures complied with the Declaration of Helsinki. In addition, the study was approved by the ethics committee of the Second Affiliated Hospital of Nantong University. Written informed consent was obtained from at least one parent or legal guardian who had been provided with information about the study, and oral consent was obtained from the participants.

In all subjects, a complete eye examination including best-corrected visual acuity (BCVA), slit-lamp examination, non-contact tonometry, and funduscopy was performed. A Snellen chart was used to record the BCVA. Detailed histories including age, sex, drug use, and systemic disease were recorded for all participants. The inclusion criteria were age younger than 18 years old; a BCVA above 10/10 (on the Snellen scale); an intraocular pressure (IOP) ≤ 21 mmHg; a cup-to-disc (*C*/*D*) ratio of ≤0.4; and a *C*/*D* ratio asymmetry of ≤0.2 between both eyes. We excluded individuals with other ocular diseases besides ametropia, binocular vision abnormalities, crystalline lens opacities, a history of corneal contact lens usage, previous use of atropine or other types of cycloplegias, allergy to atropine, poor systemic health, unwillingness to participate, or active ocular infection, which is a contraindication for UBM examination.

Ninety-three children, 7 to 17 years of age with all types of refractive error, were enrolled in this study. To account for variability in procedures performed by different observers and to improve reproducibility, in UBM examination, the anterior segment was imaged with the same device (QUANTEL AVISO), and a 50 MHz probe was used by the same experienced examiner (Li Jun Jie). All measurements were taken before and after cycloplegia. For this purpose, 1% atropine sulfate ointment was used two times per day for 7 days in the right eye. Only the right eye was examined in the study, and each participant had UBM performed 2 times separated by a 7-day interval. We planned to terminate the study and arrange for appropriate treatment if a participant had a serious allergic reaction.

UBM examinations were performed on participants lying in a supine position in a dimly lit room (illumination approximately 60–70 lux, measured with a luminance meter). An eye cup containing sterile water for injection was mounted on the globe after topical anesthesia (oxybuprocaine) was used, and the transducer was applied gently with just enough pressure to prevent leakage of fluid. First, the probe was scanned in a direction perpendicular to the center of the pupil (panoramic scanning image). When the following requirements were met, the image was considered to provide a valid measurement [15]: (1) the image was symmetrically aligned with the iris and balanced horizontally; (2) the cornea and anterior capsules were balanced over a theoretical vertical central line; and (3) a clear sulcus was present bilaterally. Then, radial scan images at the 12 (superior), 3 (nasal), 6 (inferior), and 9 (temporal) o'clock positions centered over the limbus and ciliary body region to be examined were obtained. Efforts were made to ensure that the probe was perpendicular to the limbus of the scanned quadrant, and the images provided a clear view of the scleral spur, angle, ciliary body, iris, and anterior surface of the lens. If the participant was uncomfortable or unwilling, the examination was stopped immediately.

After image capture, the most illustrative and accurate frames from the same quadrant were chosen to study anterior segment biometric characteristics. The system's integrated measurement cursors were used by the examiner (Xiong Yaojia) to obtain the measurements. Each measurement was performed three times, and the average value was taken.

**Figure 1:**
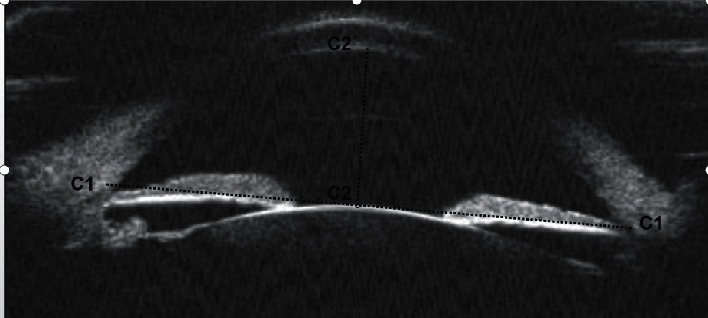
ACW (C1) is defined as the horizontal distance from one scleral spur to another; ACD (C2) is defined as the distance from the corneal endothelium to the anterior surface of the lens.

**Figure 2:**
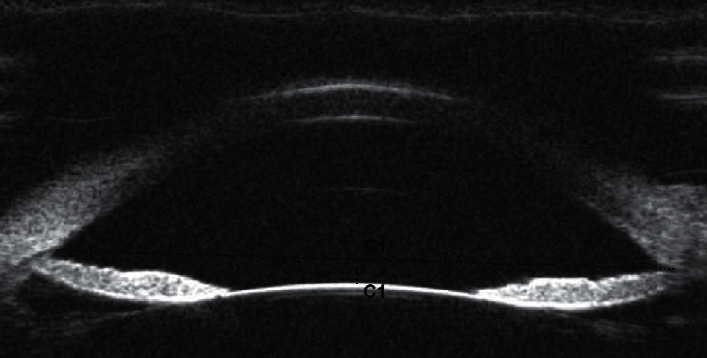
LV (C1) is the perpendicular distance between the anterior pole of the crystalline lens and the horizontal line joining the two scleral spurs.

The UBM parameters of anterior chamber width (ACW), anterior chamber depth (ACD), and lens vault (LV), as defined by Pavlin et al. [16] and some recent studies [17, 18], were assessed from the panoramic (angle to angle) images in Figures 1 and 2.

**Figure 3:**
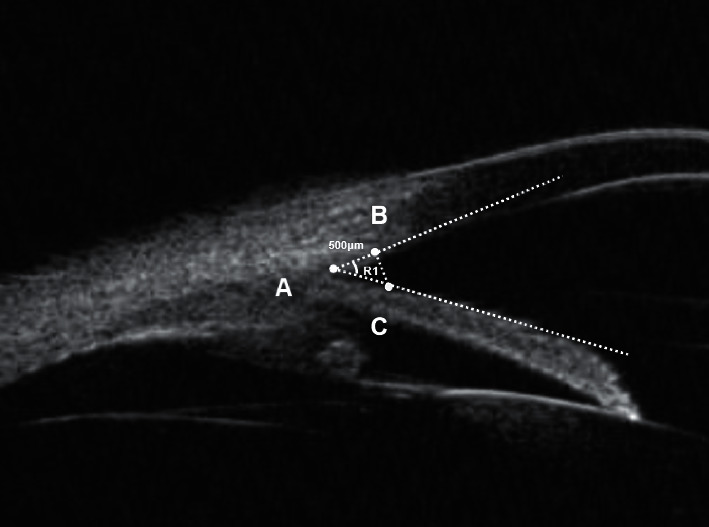
TIA (R1) is measured between the axis of the iris and a line connecting the scleral spur (point A) to the inner corneal surface with a distance of 500 *μ*m (point B). AOD 500 (segment B to C) is the distance between the posterior corneal surface and the anterior iris surface, measured in a line perpendicular to the trabecular meshwork at 500 mm from the scleral spur.

**Figure 4:**
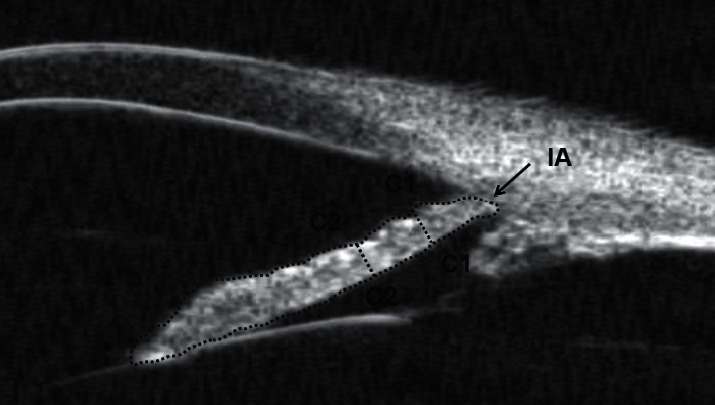
IT750 (C1) is the iris thickness at 750 *μ*m from the scleral spur; IT1500 (C2) is the iris thickness at 1500 *μ*m from the scleral spur; IA was calculated as the entire cross-sectional area of the iris from the scleral spur to the pupil.

**Figure 5:**
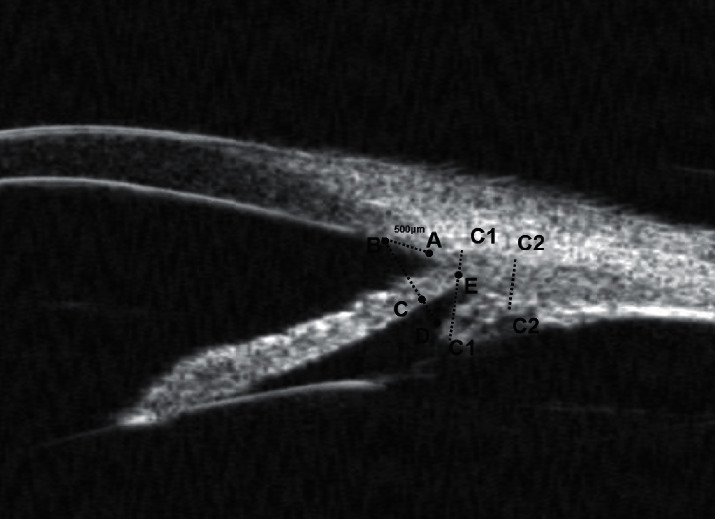
Point A represents scleral processes; point E represents the root of the iris; CBT0 (C1) is the thickness of the ciliary body at the point of the root of the iris; CMTmax (C2) is the thickest location of the ciliary muscle; TCPD (segment B to D) is measured in a line extending from the corneal endothelium at 500 *μ*m from the scleral spur perpendicularly through the iris to the most anterior ciliary processes; ICPD (segment C to D) is measured from the posterior iris surface to the ciliary process along the same line as the TCPD.

**Figure 6:**
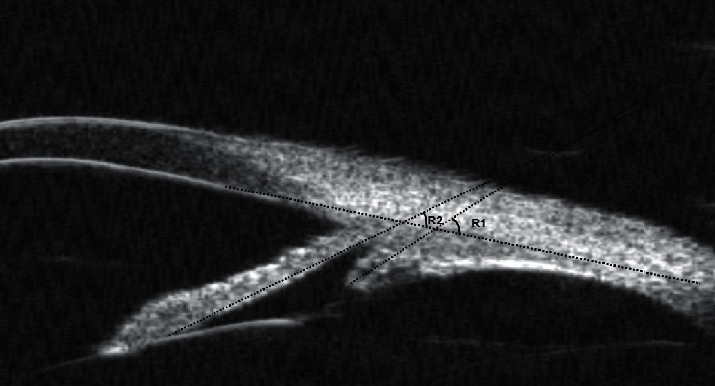
SCPA (R1) is measured between the tangent to the scleral surface and the axis of the ciliary process; and SIA (R2) is measured between the tangent to the scleral surface and the long axis of the iris.

**Figure 7:**
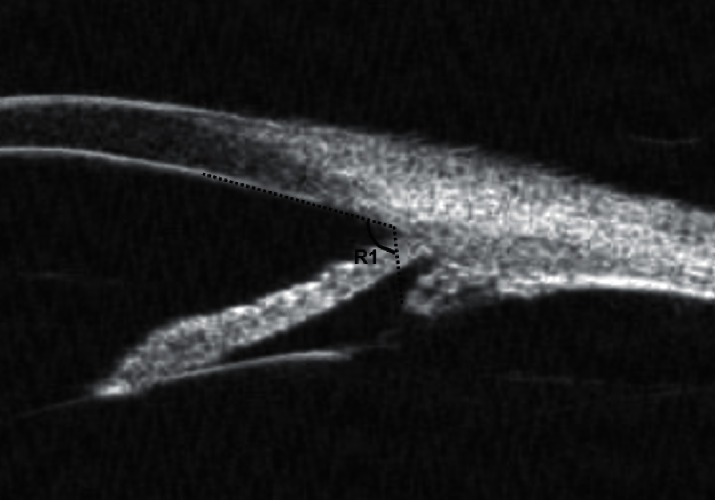
TCA (R1) is defined as the angle between the posterior corneal surface and the anterior surface of the ciliary body.

Then, other parameters defined by Pavlin et al. [16] were acquired form the radial cross section. The UBM parameters trabecular meshwork-iris angle (TIA) and angle opening distance at 500 *μ*m from the scleral spur (AOD500) are assessed in Figure 3. The UBM parameters iris thickness at 750 *μ*m (IT750), iris thickness at 1500 *μ*m (IT1500), and iris area (IA) are assessed in Figure 4. The thickness of the ciliary body at the point of the root of the iris (CBT0), maximum ciliary muscle thickness (CMTmax), trabecular-ciliary process distance (TCPD), and iris-ciliary process distance (ICPD) were assessed, as shown in Figure 5. The UBM parameters sclera-iris angle (SIA) and sclera-ciliary process angle (SCPA) are assessed in Figure 6. The trabecular-ciliary angle (TCA) is assessed in Figure 7. All measurements were performed between 2 p.m. and 4 p.m. The contrast and noise were adjusted to ensure clear images.

### 2.1. Statistical Analysis

Data were analyzed in SPSS analysis software (version 22; SPSS Inc., Chicago, IL, USA). The Kolmogorov–Smirnov test was used to test the normality of the parameters. A paired *t*-test was used to compare the parameters before and after cycloplegia. Univariate regression was conducted with changes in ΔTIA as the dependent variable and included the effects of age, sex, AOD500, ACD, LV, ACW, IT750, IT1500, IA, CBT0, CMTmax, TCA, TCPD, ICPD, SIA, SCPA, TIA, the change in AOD500 (ΔAOD500), the change in ACD (ΔACD), the change in LV (ΔLV), the change in ACW (ΔACW), the change in IT750 (ΔIT750), the change in IT1500 (ΔIT1500), the change in IA (ΔIA), the change in CBT0 (ΔCBT0), the change in CMTmax (ΔCMTmax), the change in TCA (ΔTCA), the change in TCPD (ΔTCPD), the change in ICPD (ΔICPD), the change in SIA (ΔSIA), and the change in SCPA (ΔSCPA). All results with *P* < 0.05 were considered to indicate statistical significance.

## 3. Results

### 3.1. Measurements before and after Cycloplegia

Of the 93 children who were enrolled in this study, four were excluded because of IOP measurements greater than 21 mmHg; three were excluded because of other ocular diseases; and eight were excluded because of poor coordination or unwillingness to continue to participate.

A total of 78 children who met the inclusion criteria completed the first and second examinations. However, 20 children were excluded because good UBM images from the same quadrant could not be collected. The mean age was 10.79 ± 2.53, with a range of 7–17 years. Finally, 21 boys and 37 girls were included in the statistical analysis. All participants were Chinese. Except for ΔIA (*P*=0.032) and ΔLV (*P*=0.022), the parameters and their differences before and after cycloplegia conformed to a normal distribution. The age distribution did not conform to a normal distribution (*P* < 0.01).  Table 1 summarizes the biometric characteristics of all patients before and after the use of atropine. The anterior chamber angle widened significantly after cycloplegia, the TIA increased from 38.3° ± 7.59° to 51.44° ± 9.33° (*P* < 0.05), and the AOD500 increased from 0.63 mm ± 0.19 mm to 0.9 mm ± 0.25 mm (*P* < 0.05). After cycloplegia, the depth of the anterior chamber increased from 3.16 mm ± 0.23 mm to 3.31 mm ± 0.21 mm (*P* < 0.05), and the change in LV from −0.1 mm ± 0.17 mm to −0.34 mm ± 0.17 mm (*P* < 0.05) suggested that the lens moved backward significantly. After cycloplegia, the thickness of the iris increased significantly, the IT750 increased from 0.44 mm ± 0.1 mm to 0.55 mm ± 0.1 mm (*P* < 0.05), and the IT1500 increased from 0.48 mm ± 0.07 mm to 0.58 mm ± 0.08 mm (*P* < 0.05). The iris cross-sectional area decreased significantly, and the IA decreased from 1.87 mm^2^ ± 0.27 mm^2^ to 1.36 mm^2^ ± 0.21 mm^2^ (*P* < 0.05). The iris showed a distinct backward rotation, and the SIA increased from 45.58° ± 6.96° to 61.99° ± 10.62° (*P* < 0.05). After cycloplegia, the thickness of the ciliary body in the iris root did not change significantly, and the CMTmax decreased from 0.68 ± 0.08 to 0.58 ± 0.08 (*P* < 0.05). The ciliary process also showed a significant backward rotation, the TCA increased from 70.54° ± 13.02° to 86.47° ± 14.39° (*P* < 0.05), and the TCPD increased from 1.06 mm ± 0.15 mm to 1.18 mm ± 0.17 mm (*P* < 0.05).

### 3.2. Univariate Linear Regression Modeling

**Figure 8:**
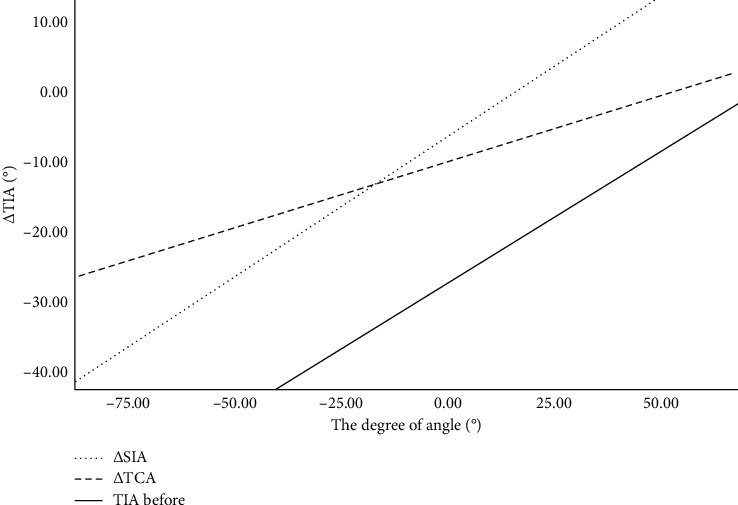
ΔTCA (°), ΔSIA (°), and TIA (°) before cycloplegia versus ΔTIA (°).

Both ΔTIA and ΔAOD500 reflect the change in the anterior chamber angle in different ways: TIA directly reflects the opening angle of the anterior chamber angle, whereas AOD500 reflects the opening degree of the anterior chamber angle through a specific measurement distance. To more intuitively demonstrate the change in the anterior chamber angle, we used the parameter ΔTIA in a further study with univariate analysis. The results are presented in Table 2. A greater ΔTIA was significantly associated with a greater ΔTCA (*P*=0.004), ΔSIA (*P* < 0.001), ΔAOD500 (*P* < 0.001), and TIA before cycloplegia (*P*=0.01). The relevant scatter diagrams are shown in Figure 8.

## 4. Discussion

UBM is widely used to evaluate the anterior segment. The development of new imaging techniques for the anterior segment has led to the emergence of new analysis methods such as optical coherence tomography [19, 20], IOLMaster [21], and Pentacam [14]. When measuring the anterior chamber angle, some studies have suggested that the results of these new methods are similar to those of UBM [22, 23]. UBM is also believed to be more accurate in measuring certain anterior segment parameters [24], and it is the only imaging technique that can be used for all anterior segment structures, particularly the ciliary body [25]. Given the possible effects of atropine on ciliary muscle, the use of UBM is appropriate.

The importance of this study is that, in China, atropine is widely used in the diagnosis and treatment of myopia. Few studies have comprehensively investigated the structure of the entire anterior segment of the eye before and after atropine administration. The merit of this study is that it provides information on how the anterior segment behaves in response to atropine without the confounding factor of cataracts. Our results showed that, after cycloplegia induced by atropine, the ACD became deeper, from 3.16 ± 0.23 mm to 3.31 ± 0.21 mm, the SIA increased from 45.58° ± 6.96° to 61.99° ± 10.62°, and the SCPA increased from 52.22° ± 7.81° to 58.93° ± 7.18°; these findings were consistent with those of previous studies [12, 13, 26]. One of the most important findings in this study was the increase in TIA, which indicated that the anterior chamber angle widened after cycloplegia from 38.3° ± 7.59° to 51.44 ± 9.33°. In previous similar studies, other ciliary muscle paralysis drugs have generally been used instead of atropine, and in adults, the anterior chamber angle tends to be shallower with cycloplegia. Arici et al. [27] have shown that the use of 1% cyclopentolate in young adults, as measured by Pentacam, resulted in an anterior chamber angle decrease from 38.09° ± 8.13° to 33.95° ± 10.14°. Similar results have been obtained with the use of 1% tropicamide by Palamar et al. [28]. In their research on adults, the mean Pentacam ACA measurements for the left eyes were 38.85° ± 6.05° pre-tropicamide and 33.39° ± 9.77° post-tropicamide. However, measurement of the anterior chamber angle after ciliary muscle palsy in children is controversial. Tsai et al. [12] have investigated the changes in the anterior chamber configuration in children (6–13 years of age) after diagnostic mydriasis and found that, with the use of 1% tropicamide, the mean ACA increased from 46.33° ± 7.01° to 52.65° ± 6.21°. Ying et al. [13] have observed that, with the use of 0.5% tropicamide, the Pentacam ΔACA in the hyperopic group slightly increased, whereas that in the emmetropic and myopic groups decreased in children (6–13 years of age). Palamar et al. [14] have reported that the differences between pre- and post-cycloplegia ACA measurements after 1% cyclopentolate hydrochloride instillation were not statistically significant in their study. These inconsistencies among studies can be explained as follows. First, the drugs used and the concentrations of drugs differed, thus probably causing different degrees of cycloplegia and pupil dilation and resulting in different changes in anterior lens displacement. Second, the baseline anterior segment parameters were different. Third, the ages of the study populations were different. Anterior chamber narrowing appears to occur after ciliary muscle paralysis in adults.

The univariate analysis in our study indicated that greater ΔTIA values were mainly associated with greater ΔTCA and ΔSIA values. Thus, the posterior rotation of the iris plane and ciliary processes caused by cycloplegia was significantly associated with the widening of the anterior chamber angle. In this study, the TCPD increased from 1.06 ± 0.15 mm to 1.18 ± 0.17 mm (*P* < 0.05), thus indicating that the ciliary process had significant retroversion after cycloplegia, but the ICPD showed no significant change (*P*=0.14), thus indicating that the degree of retroversion of the iris after cycloplegia was consistent with that of the ciliary process. We speculate that this interesting balance is also one of the factors ensuring the widening of the anterior chamber angle. If the degree of posterior rotation of the ciliary body were less than the degree of posterior rotation of the iris, relative anterior rotation of the ciliary body would occur, thus resulting in a relative narrowing of the anterior chamber angle. Atropine is a nonspecific muscarinic antagonist [29], and some studies have shown that atropine can produce greater cycloplegia than other drugs [30–32]. We speculate that this aspect may explain why our results indicated greater anterior chamber angle widening than that in previous studies.

Despite some controversy, several previous studies have shown that atropine use in adults may lead to elevated IOP. Systemic atropine may increase IOP by more than 6 mmHg in 8% of normal adults [33]. Harris have shown that atropine may lead to elevations in IOP up to 23% in proven open-angle glaucoma but only 2% in an apparently normal population [34]. Furthermore, an increase in IOP has been observed after local application of other cycloplegic and mydriatic drugs [35, 36] in eyes with open or closed iridocorneal angles in adults [34, 37, 38]. Mydriatic or cycloplegic agents can increase IOP, an effect that might be associated with decreased aqueous outflow, owing to the blockage of the anterior chamber angle of the dilated and immobile iris [39]. In children, however, recent studies have detected no significant changes in IOP before and after atropine administration. Wu et al. [40] have analyzed 621 children with atropine doses ranging from 0.1% to 1%, and statistical analyses did not indicate any relationship between the dose or duration of atropine therapy and the risk of elevated IOP. A prospective study by Lee et al. [41] has revealed similar results. Therefore, the use of atropine in children with normal routine ophthalmic examination results does not raise concerns regarding elevated intraocular pressure.

Recent reports support that the dynamic responses of the eye during pupil dilation, particularly iris changes, have a major role in angle-closure pathogenesis [42]. Thickening of the iris root under dark conditions has been demonstrated to be associated with angle closure [43]. Moreover, iris thickening after cycloplegia is believed to decrease the anterior chamber angle [26]. In this study, we observed an IT750 increase from 0.44 ± 0.1 mm to 0.55 ± 0.1 mm, an IT1500 increase from 0.48 ± 0.07 mm to 0.58 ± 0.08 mm, and an IA decrease from 1.87 ± 0.27 mm^2^ to 1.36 ± 0.21 mm^2^. These findings were consistent with those of previous studies [44, 45]. However, in this study, we confirmed that these changes were not associated with changes in the anterior chamber angle. We speculate that although the root of the iris was thickened, the iris plane was clearly back rotated, thus preventing the thickening of the iris from narrowing the anterior chamber angle.

To analyze the changes in ciliary muscle with accommodation, investigators have used 1% cyclopentolate or 1% tropicamide for ciliary muscle paralysis [46, 47]. Previous studies have confirmed that the ciliary muscle thickness at CMTmax increases with accommodation, and we verified that the ciliary muscle thickness at CMTmax decreased with cycloplegia. In this article, with the use of atropine, the CMTmax decreased from 0.68 ± 0.08 mm to 0.58 ± 0.08 mm.

LV has recently been identified as a novel risk factor for angle closure in Chinese Singaporeans and in a Japanese population [18, 48]. In a study by Nongpiur et al. [18], only the LV was found to be independently associated with angle closure, after multivariate analysis adjusted for age, sex, ACD, LT, and RLP. In that study, the authors measured an LV of 0.316 ± 0.272 mm at 54.2 ± 7.9 years of age in normal eyes. To our knowledge, this study is the first to measure LV in children; the measured value was −0.1 ± 0.17 mm before cycloplegia. Interestingly, the mean value of LV is positive in elderly people and negative in children. We speculate that this difference may be one reason for the low risk of closure of the anterior chamber angle in children and may be associated with the changes in the lens and vitreous body with age, although these possibilities require further study.

### 4.1. Study Limitations

Our study has several limitations. First, the posterior capsule of the lens is often unclear in UBM measurements with a 50 MHz probe, and therefore the change in lens thickness cannot be measured accurately. Although we attempted to use the LV to represent the changes in the lens, the relationship between the anterior chamber angle and lens thickness still requires further study. Second, factors such as refraction and axial length were not evaluated. Third, the study sample was not population based; instead, it was a hospital-based sample of school-age children. Changes in the anterior segment in the eyes at all ages warrant further study. Finally, in the study of children's anterior structure, several simpler non-contact examinations, such as AS-OCT, may be promising and warrant further study.

In conclusion, we found that 1% atropine causes statistically significant changes in various anterior segment parameters. Compared with findings from previous studies, this study indicated that some changes were different from those in adults. The change in anterior chamber angle is associated with ΔTCA, ΔSIA, and TIA before cycloplegia.

## Figures and Tables

**Table 1:** The ocular biometry measurements obtained by using UBM with and without cycloplegia.

Parameter	Mean ± SD			*P* value
	Before cycloplegia	After cycloplegia	Mean difference ± SD	
TIA (°)	38.3 ± 7.59	51.44 ± 9.33	−13.15 ± 8.53	<0.05
AOD500 (mm)	0.63 ± 0.19	0.9 ± 0.25	−0.28 ± 0.16	<0.05
ACD (mm)	3.16 ± 0.23	3.31 ± 0.21	−0.16 ± 0.14	<0.05
LV (mm)	−0.1 ± 0.17	−0.34 ± 0.17	0.24 ± 0.16	<0.05
ACW (mm)	12.17 ± 0.68	12.03 ± 0.64	0.14 ± 0.64	=0.1
IT750 (mm)	0.44 ± 0.1	0.55 ± 0.1	−0.11 ± 0.11	<0.05
IT1500 (mm)	0.48 ± 0.07	0.58 ± 0.08	−0.1 ± 0.09	<0.05
IA (mm^2^)	1.87 ± 0.27	1.36 ± 0.21	0.52 ± 0.29	<0.05
CBT0 (mm)	1.22 ± 0.11	1.21 ± 0.16	0.01 ± 0.16	=0.52
CMTmax (mm)	0.68 ± 0.08	0.58 ± 0.08	0.1 ± 0.08	<0.05
TCA (°)	70.54 ± 13.02	86.47 ± 14.39	−15.93 ± 16.79	<0.05
TCPD (mm)	1.06 ± 0.15	1.18 ± 0.17	−0.12 ± 0.19	<0.05
ICPD (mm)	0.2 ± 0.13	0.17 ± 0.11	0.03 ± 0.15	=0.14
SIA (°)	45.58 ± 6.96	61.99 ± 10.62	−16.41 ± 9.51	<0.05
SCPA (°)	52.22 ± 7.81	58.93 ± 7.18	−6.71 ± 8.93	<0.05

**Table 2:** Univariate analysis of the associations between ΔTIA and the ocular and general parameters.

Variables	Univariate analysis		
	Regression coefficient (95% confidence interval)	*R* ^2^	*P* value
Age	−0.324 (−1.049 to 0.463)	−0.008	0.578
Sex	−3.256 (−15.717 to 0.085)	0.017	0.164
ΔAOD500 (mm)	26.64 (14.871 to 38.146)	0.251	<0.05
ΔACD (mm)	3.007 (−13.465 to 19.478)	−0.015	0.716
ΔLV (mm)	8.448 (−2.589 to 19.741)	0.009	0.226
ΔACW (mm)	0.766 (−2.881 to 3.766)	−0.014	0.665
ΔIT750 (mm)	1.743 (−17.353 to 21.07)	−0.017	0.871
ΔIT1500 (mm)	−9.287 (−35.575 to 18.34)	−0.008	0.467
ΔIA (mm67)	−2.34 (−8.122 to 5.676)	−0.012	0.557
ΔCBT0 (mm)	5.693 (−7.496 to 18.84)	−0.007	0.436
ΔCMTmax (mm)	8.252 (−19.678 to 37.582)	0.012	0.574
ΔTCA (CA)	0.189 (0.057 to 0.344)	0.123	0.004
ΔTCPD (mm)	−4.274 (−14.436 to 6.66)	−0.009	0.484
ΔICPD (mm)	−3.513 (−17.697 to 12.185)	−0.014	0.641
ΔSIA (IA)	0.4 (0.204 to 0.591)	0.185	<0.05
ΔSCPA (CP)	0.071 (−0.264 to 0.331)	−0.012	0.579
AOD500 (mm)	−2.007 (−13.807 to 11.251)	−0.016	0.735
ACD (mm)	−6.162 (−16.044 to 3.72)	0.01	0.217
LV (mm)	5.148 (−8.016 to 18.312)	−0.007	0.437
ACW (mm)	1.018 (−2.317 to 4.353)	−0.011	0.543
IT750 (mm)	10.254 (−13.994 to 33.17)	−0.003	0.371
IT1500 (mm)	−0.945 (−36.492 to 35.326)	−0.018	0.954
IA (mm54)	−0.745 (−9.2 to 7.709)	−0.017	0.86
CBT0 (mm)	8.709 (−13.341 to 32.765)	−0.005	0.399
CMTmax (mm)	3.865 (−23.512 to 30.92)	−0.016	0.785
TCA (CA)	0.114 (−0.046 to 0.268)	0.013	0.193
TCPD (mm)	−11.043 (−24.1 to 2.248)	0.022	0.137
ICPD (mm)	−6.593 (−21.547 to 8.072)	−0.008	0.469
SIA (IA)	−0.016 (−0.344 to 0.312)	−0.018	0.922
SCPA (CP)	−0.007 (−0.299 to 0.286)	−0.018	0.964
TIA (IA)	0.375 (0.128 to 0.631)	0.96	0.01

## Data Availability

The data used to support the findings of this study are available from the corresponding author upon request.
